# National Latinx AIDS Awareness Day — October 15, 2018

**DOI:** 10.15585/mmwr.mm6740a1

**Published:** 2018-10-12

National Latinx AIDS Awareness Day (https://www.cdc.gov/Features/LatinoAIDSAwareness), October 15, is observed each year to focus on the continuing and disproportionate impact of human immunodeficiency virus (HIV) infection and acquired immunodeficiency syndrome (AIDS) on Hispanics/Latinos in the United States. The prevalence of diagnosed HIV infection among Hispanics/Latinos is approximately twice that among non-Hispanic whites (*1*). The percentage of persons with diagnosed infection who are virally suppressed (<200 copies of HIV RNA per mL of blood) is lower among Hispanics/Latinos than among non-Hispanic whites (*2*).

An analysis of clinical outcomes among Hispanic/Latino participants in CDC’s Medical Monitoring Project (2013 and 2014 cycles) found that a significantly higher percentage of women (78%), compared with men (54%), were living in poverty (*3*). However, women and men were equally likely to have received prescriptions for antiretroviral therapy (95% versus 96%) and to have durable viral suppression (68% versus 73%) (*3*).

National Latinx AIDS Awareness Day is an opportunity to encourage increased HIV prevention activities among Hispanics/Latinos. CDC supports testing; linkage to, and engagement in, care and treatment; and other efforts to reduce the risk for acquiring or transmitting HIV infection among Hispanics/Latinos. Additional information is available at https://www.cdc.gov/hiv/group/racialethnic/hispaniclatinos/index.html.
